# Supplementary material for: Single-molecule imaging of PI(4,5)P2 and PTEN in vitro reveals a positive feedback mechanism for PTEN membrane binding
Source: Commun Biol. 2020 Feb 28;3:92. doi: 10.1038/s42003-020-0818-3 (PMC7048775; doi:10.1038/s42003-020-0818-3)
Supplement: Supplementary file 1 — Supplementary Information [file 42003_2020_818_MOESM1_ESM.pdf]

Supplementary Figures

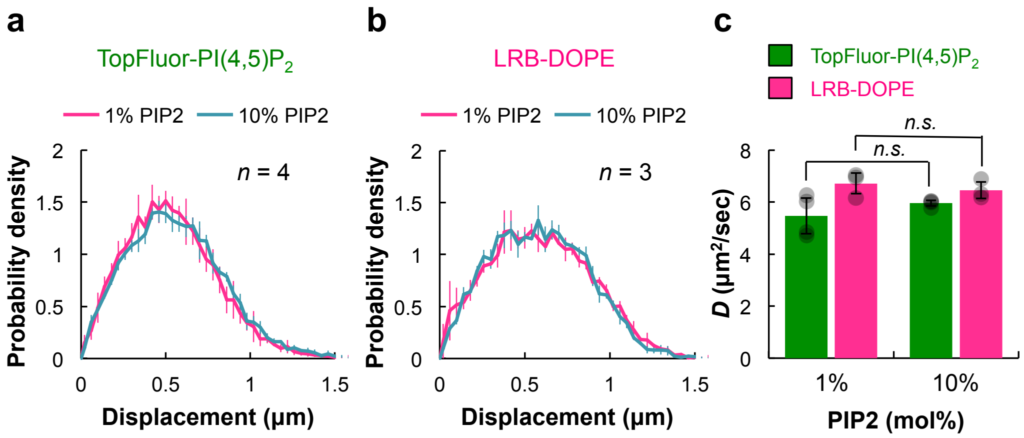

Supplementary Figure 1. Lateral diffusion of LRB-DOPE in artificial lipid bilayers. (a-b) Probability density distribution of the displacement of TopFluor-PI(4,5)P_2 and LRB-DOPE, which possesses standard dioleoyl fatty acid chains and a fluorphore incorporated into the head group where it does not significantly perturb the diffusion speed, on the lipid bilayers of 1 mol% PI(4,5)P_2 (*magenta*) or 10 mol% PI(4,5)P_2 (*blue*). (c) Average diffusion coefficients quantified in (a-b). $P = 0.30$ and $P = 0.51$ for 1 mol% versus 10 mol% PI(4,5)P_2 for TopFluor-PI(4,5)P_2 (*green*, $n = 4$ separate movies in 2 independent experiments) and LRB-DOPE (*magenta*, $n = 3$ separate movies in 3 independent experiments). P values were obtained by Welch's t test. Data are the mean ± SD.

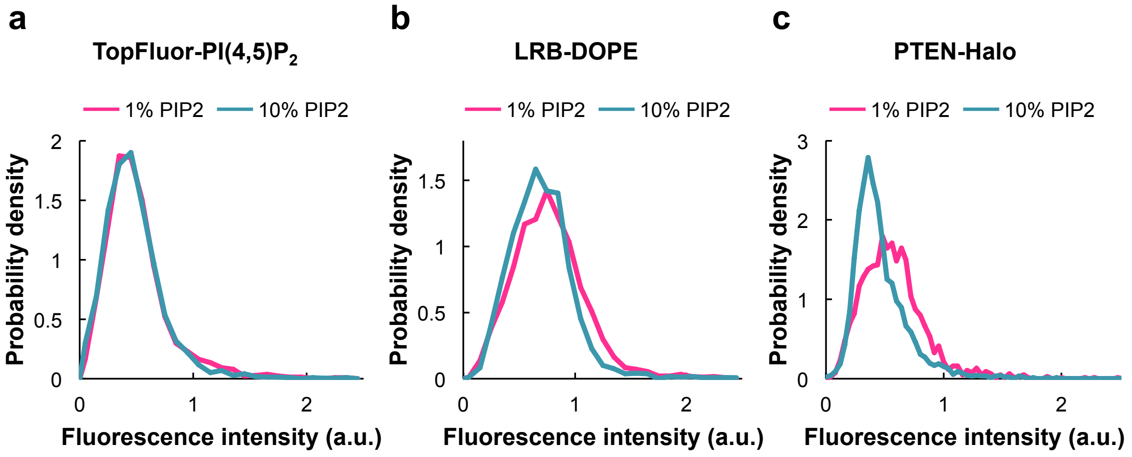

Supplementary Figure 2. Fluorescence intensity distribution of lipids and PTEN on artificial lipid bilayers. (a-c) Fluorescence intensity distribution of single molecules of TopFluor-PI(4,5)P_2, LRB-DOPE and PTEN-Halo-TMR on artificial lipid bilayers composed of 1 mol% PI(4,5)P_2 (*magenta*) or 10 mol% PI(4,5)P_2 (*blue*). All distributions were obtained from 500 molecules. Single-peaked distributions provide proof of the successful imaging of single molecules.

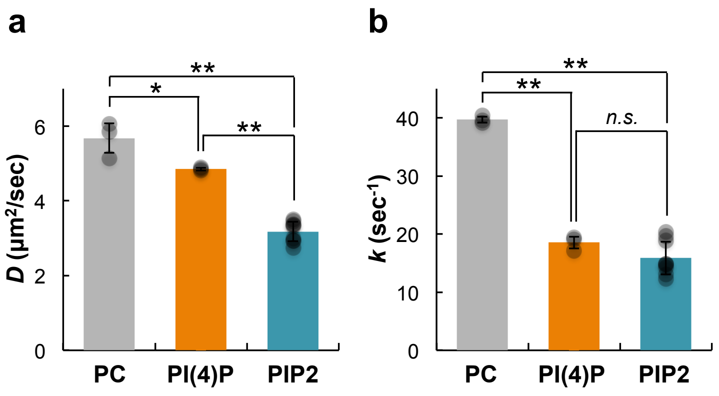

Supplementary Figure 3. Effects of PI(4)P on the membrane-binding kinetics of PTEN *in vitro*. (a) The average diffusion coefficients of PTEN-Halo on 100 mol% PC (*gray*), 10 mol% PI(4)P (oran*ge*) and 10 mol% PI(4,5)P_2 (*blue*) lipid bilayers. $P = 0.014$ for PC ($n = 3$ movies in 3 independent experiments) versus PI(4)P ($n = 3$ movies in 3 independent experiments), $P = 0.000$ for PC versus PI(4,5)P_2 ($n = 9$ movies in 3 independent experiments), $P = 0.000$ for PI(4)P versus PI(4,5)P_2 by the Tukey-Kramer test. (b) The average dissociation rate constants of PTEN-Halo on 100 mol% PC (*gray*), 10 mol% PI(4)P (oran*ge*) and 10 mol% PI(4,5)P_2 (*blue*) lipid bilayers. $P = 0.000$ for PC versus PI(4)P, $P = 0.000$ for PC versus PI(4,5)P_2, $P = 0.284$ for PI(4)P versus PI(4,5)P_2 by the Tukey-Kramer test. Data are the mean ± SD.

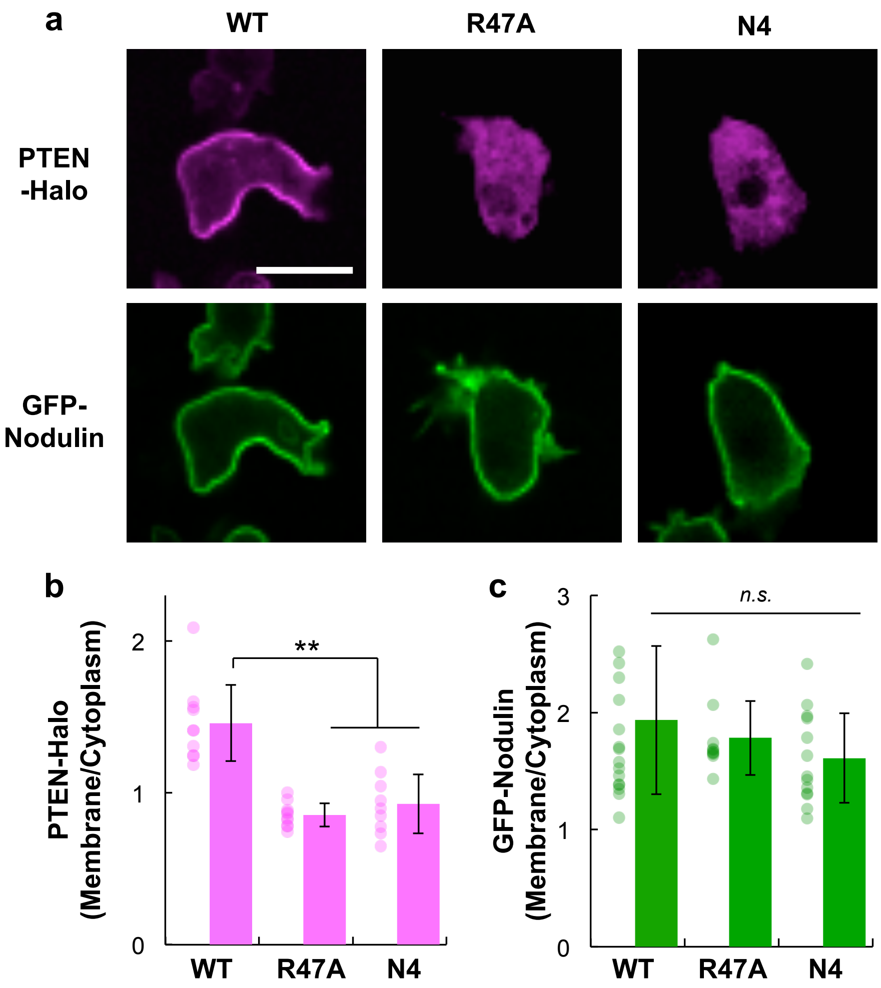

Supplementary Figure 4. PI(4,5)P_2 levels in Ax2 cells expressing recombinant PTEN-Halo. (a) Representative confocal images of *Dictyostelium discoideum* wild-type cells (Ax2) expressing PTEN-Halo labeled with TMR (*magenta*) and GFP-Nodulin (*green*). Scale bar, 10 μm. (b) Membrane accumulation of PTEN quantified by the fluorescence intensity ratio of PTEN-Halo-TMR between the membrane and cytoplasm in (a). $P = 0.000$ for $PTEN_{WT}$ ($n = 10$ cells) versus $PTEN_{R47A}$ ($n = 10$ cells), $P = 0.000$ for $PTEN_{WT}$ versus $PTEN_{N4}$ ($n = 9$ cells), $P = 0.714$ for $PTEN_{R47A}$ versus $PTEN_{N4}$ by the Tukey-Kramer test. (c) PI(4,5)P_2 density on the plasma membrane quantified by the fluorescence intensity ratio of GFP-Nodulin between the membrane and cytoplasm in (a). There was no significant difference by the Tukey-Kramer test; $P = 0.734$ for $PTEN_{WT}$ ($n = 18$ cells) versus $PTEN_{R47A}$ ($n = 10$ cells), $P = 0.207$ for $PTEN_{WT}$ versus $PTEN_{N4}$ ($n = 13$ cells), $P = 0.710$ for $PTEN_{R47A}$ versus $PTEN_{N4}$. Data are the mean ± SD.

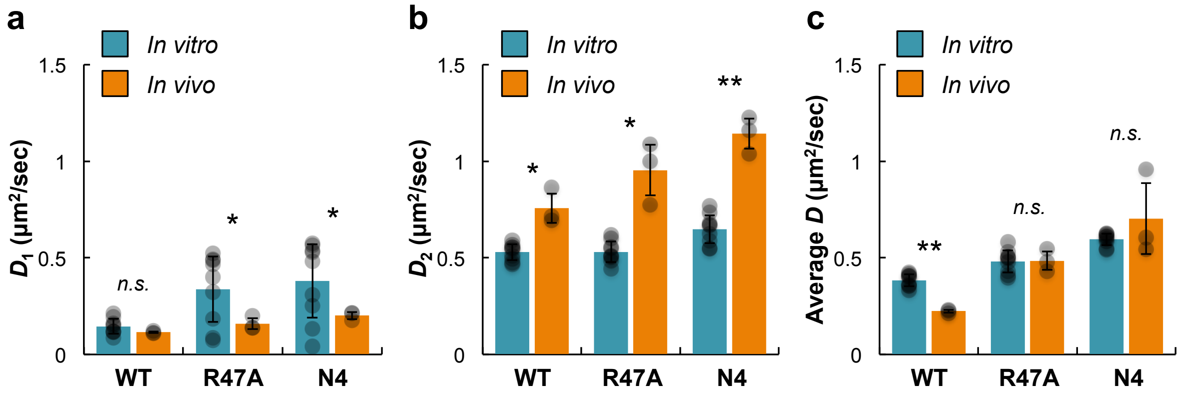

Supplementary Figure 5. PTEN diffusion coefficients *in vitro* and *in vivo*. To compare the diffusion coefficient of PTEN on artificial membrane (AM) and plasma membrane (PM), the diffusion coefficient of PTEN *in vitro* ($D_{\text{PTEN\_}in\ vitro}$) was corrected as $D_{\text{PTEN\_}in\ vitro}*D_{\text{PM}}/D_{\text{AM}}$, where D_{PM} and D_{AM} represent the diffusion coefficients of PM and AM, respectively. (a-c) Diffusion coefficients of PTEN, D_1 and D_2, for the slower and faster mobility states, respectively. *Blue* and *orange* indicate $D_{\text{PTEN\_}in\ vitro}*D_{\text{PM}}/D_{\text{AM}}$ ($n = 9$ separate movies in 3 independent experiments at 10 mol% PI(4,5)P_2) and $D_{\text{PTEN\_}in\ vivo}$, ($n = 3$ cells) respectively. Welch's t-test; comparisons were made with '*In vitro*' column for the same cell line. (a) $P = 0.055$, 0.019 and 0.030 for WT, R47A and N4, respectively. (b) $P = 0.043$, 0.041 and 0.004 for WT, R47A and N4, respectively. (c) $P = 0.000$, 0.923 and 0.498 for WT, R47A and N4, respectively. Data are the mean ± SD.

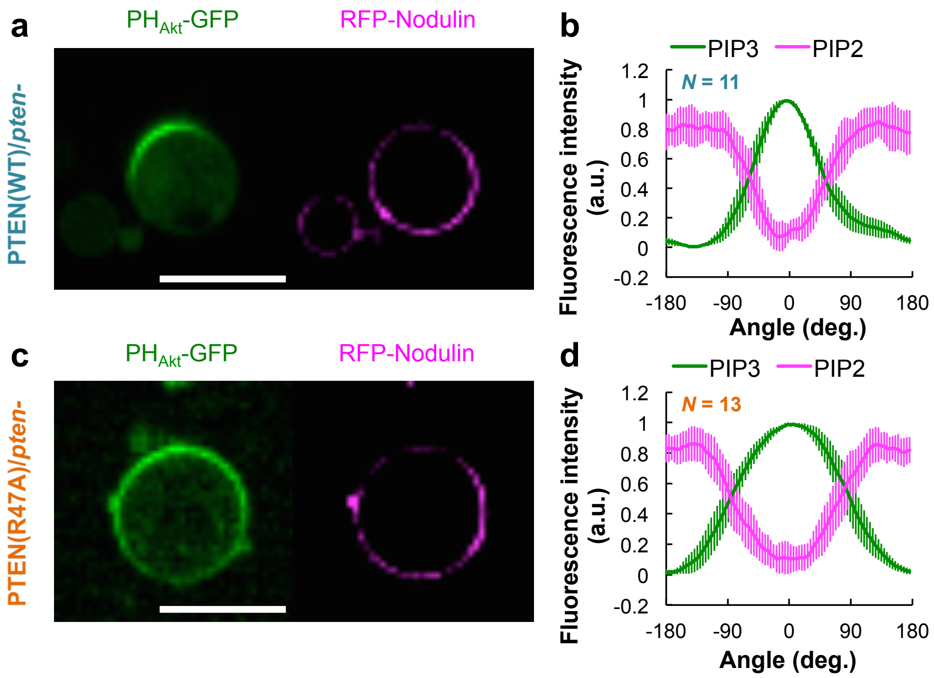

Supplementary Figure 6. Two-color simultaneous imaging of mutually exclusive localization patterns of PI(3,4,5)P_3 and PI(4,5)P_2. (a, c) A representative snapshot image of $PH_{Akt/PKB}$-EGFP (PI(3,4,5)P_3 reporter, *green*) and RFP-Nodulin (PI(4,5)P_2 reporter, *magenta*) co-expressed in PTEN-Halo/*pten-* (a) or $PTEN_{R47A}$-Halo/*pten-* (c). Scale bar, 10 μm. (b, d) Average fluorescence intensity distribution of $PH_{Akt/PKB}$-EGFP (*green*) and RFP-Nodulin (*magenta*) on the plasma membrane of *pten*-null cells expressing PTEN (b, n = 11 cells) and $PTEN_{R47A}$ (d, n = 13 cells) quantified along the cell periphery. The fluorescence intensity is shown as min-max normalization. Data are the mean ± SD.

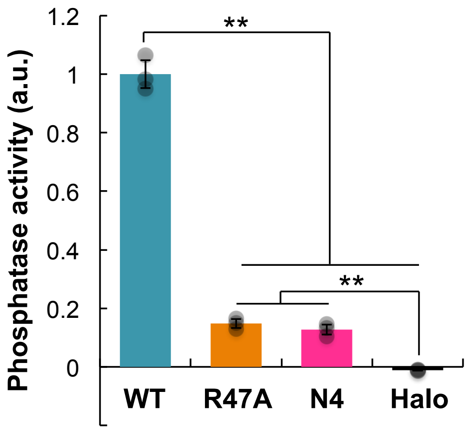

Supplementary Figure 7. Phosphatase activity of recombinant PTEN *in vitro*. By an *in vitro* assay using malachite green reagent, the PI(3,4,5)P_3 dephosphorylation activity of each recombinant PTEN-Halo and Halo-tag was relatively compared. The values were normalized by the average absorbance of wild-type PTEN-Halo at 620 nm. P = 0.000 for $PTEN_{WT}$ versus $PTEN_{R47A}$ or $PTEN_{N4}$ or Halo, P = 0.864 for $PTEN_{R47A}$ versus $PTEN_{N4}$, P = 0.001 for $PTEN_{R47A}$ versus Halo, P = 0.003 for $PTEN_{N4}$ versus Halo by the Tukey-Kramer test. Data are the mean ± SD from 3 independent experiments.

Supplementary Table

Supplementary Table 1. Primer sequences of PTEN mutants.

	Name	Primer sequence
Primer 1	*Xba*I (forward)	GAGCGTCGAGTCTAGAGCTAGATAAAA
Primer 2	*Spe*I (reverse)	CTAGCAGATCACTAGTTAATGATGATGATGA
Primer 3	R47A (forward)	GTTGAAGGTGTTTTTGCAAATCCAATGAA
	N4 (forward)	GTCTCTGCACAAGCAGCTGCATATCAAAAAAATGG TTACG
Primer 4	R47A (reverse)	ATCTTTCATTGGATTTGCAAAAACACCTTC
	N4 (reverse)	TTGATATGCAGCTGCTTGTGCAGAGACTGCAACTC TTAATAATAA
