# Supplementary material for: Single-molecule imaging of PI(4,5)P2 and PTEN in vitro reveals a positive feedback mechanism for PTEN membrane binding
Source: Commun Biol. 2020 Feb 28;3:92. doi: 10.1038/s42003-020-0818-3 (PMC7048775; doi:10.1038/s42003-020-0818-3)
Supplement: Supplementary file 8 — Description of Additional Supplementary items [file 42003_2020_818_MOESM8_ESM.pdf]

Description of Supplementary Movies

File name: Supplementary Movie 1

Description: TopFluor PI(4,5)P_2 in artificial lipid bilayers composed of DOPC and PI(4,5)P_2 (TIRF). Time-lapse TIRF images show single molecules of TopFluor PI(4,5)P_2 in 1 mol% (left), 10 mol% (center) and 20 mol% (right) PI(4,5)P_2 lipid bilayers, corresponding to Fig. 1, Supplementary Figure 1 and Supplementary Figure 2. Images are shown at 16.5 msec-intervals for 5 sec. Scale bar, 10 μm.

File name: Supplementary Movie 2

Description: Wild-type PTEN-Halo-TMR bound to artificial lipid bilayers composed of DOPC and PI(4,5)P_2 (TIRF). Time-lapse TIRF images show 10 mol% PI(4,5)P_2 bilayers in buffer (left), and 1 mol% (center) and 10 mol% (right) PI(4,5)P_2 bilayers in PTEN-Halo-TMR containing buffer, corresponding to Fig. 2A-C. Images are shown at 16.5 msec-intervals for 5 sec. Scale bar, 10 μm.

File name: Supplementary Movie 3

Description: Wild-type and mutant PTEN-Halo-TMR bound to 10 mol% PI(4,5)P_2 lipid bilayers (TIRF). Time-lapse TIRF images show single molecules of PTEN-Halo-TMR (left), $PTEN_{R47A}$-Halo-TMR (center) and $PTEN_{N4}$-Halo-TMR (right), corresponding to Fig. 3. Images are shown at 16.5 msec-intervals for 5 sec. Scale bar, 10 μm.

File name: Supplementary Movie 4

Description: Wild-type and mutant PTEN-Halo-TMR bound to the plasma membrane of Ax2 cells (TIRF). Time-lapse TIRF images show single molecules of PTEN-Halo-TMR (left), $PTEN_{R47A}$-Halo-TMR (center) and $PTEN_{N4}$-Halo-TMR (right) on the plasma membrane of Ax2 cells, corresponding to Fig. 4. Images are shown at 16.5 msec-intervals for 5 sec. Scale bar, 5 μm.

Description of Supplementary Data

File name: Supplementary Data 1

Description: Source data for Figure 1.

File name: Supplementary Data 2

Description: Source data for Figure 2-3.

File name: Supplementary Data 3

Description: Source data for Figure 4.

File name: Supplementary Data 4

Description: Source data for Figure 5.

File name: Supplementary Data 5

Description: Source data for Figure 6.

File name: Supplementary Data 6

Description: Source data for Supplementary Table 1.
